# The feasibility of ^18^F-FDG PET/CT for predicting pathologic risk status in early-stage uterine cervical squamous cancer

**DOI:** 10.1186/s40644-020-00340-z

**Published:** 2020-09-10

**Authors:** Shuai Liu, Lingfang Xia, Ziyi Yang, Huijuan Ge, Chunmei Wang, Herong Pan, Shaoli Song, Zhengrong Zhou

**Affiliations:** 1 Department of Nuclear Medicine, Fudan University Shanghai Cancer Center, No. 270, Dong’an Road, Xuhui District, Shanghai, 20032 China; 2 Department of Oncology, Shanghai Medical College, Fudan University, Shanghai, China; 3 Center for Biomedical Imaging, Fudan University, Shanghai, China; 4 Shanghai Engineering Research Center of Molecular Imaging Probes, Shanghai, China; 5 Department of Gynecological Oncology, Fudan University Shanghai Cancer Center, Shanghai, China; 6 Department of Pathology, Fudan University Shanghai Cancer Center, Shanghai, China; 7 Department of Radiology, Fudan University Shanghai Cancer Center, 270 Dong’an Road, Shanghai, 20032 China

**Keywords:** ^18^F-FDG, Surgical pathology, Risk factor, Uterine cervical cancer

## Abstract

**Background:**

Postoperative pathologic risk factors (PRFs) could increase the recurrence rate in early-stage uterine cervical squamous cancer (ECSC). Our study intended to explore the efficiency of ^18^F-FDG PET/CT for assessing the pathologic risk status (PRS) in ECSC patients.

**Methods:**

This retrospective study was performed in 240 ECSC patients with stage IA2-IIA2 (FIGO 2009), who underwent preoperative PET/CT scans and subsequent radical surgery between January 2010 and July 2015. Intermediate-risk (tumour diameter ≥ 4 cm, stromal invasion depth ≥ 1/2, lymphovascular space invasion (LVSI)), and high-risk factors (parametria involvement, positive surgery margin, pelvic lymph node metastasis) were confirmed by postoperative pathology. Patients with none of these PRFs were at a low risk for relapse. One of these PRFs was defined as positive risk. The relationship between each PRF and ^18^F-FDG uptake was analysed by t-test. Chi-square tests and logistic regression analyses were used to determine the efficiency of PET/CT parameters for assessing the PRS. The area under the curve (AUC) was used as an indicator for predictive efficiency.

**Results:**

Patients with higher SUVmax (*p* < 0.001), MTV (*p* < 0.001) and TLG (*p* < 0.001) had larger tumour sizes and deeper stromal invasion. Further multivariate analyses showed SUVmax and TLG were independent predictors for positive- and intermediate-risk status. In high-risk group, MTV and TLG were associated with pelvic lymph node metastasis and parametria involvement. However, only MTV was a significant indicator.

**Conclusions:**

Preoperative ^18^F-FDG PET/CT had an independent predictive value for PRS in ECSC.

## Background

Uterine cervical cancer is one of the most common leading causes of death among females [1]. The preferred treatment for clinical early-stage patients is radical surgery with/without individualized adjuvant treatment. Postoperative pathologic risk factors (PRFs) determined whether adjuvant chemo-radiotherapy is given. Cervical tumour diameter ≥ 4 cm, stromal invasion depth ≥ 1/2, and lymphovascular space invasion (LVSI) were intermediate-risk factors, and parametria involvement, positive surgery margin, and pelvic lymph node metastasis were high-risk factors in the national comprehensive cancer network (NCCN) and the international federation of gynaecology and obstetrics (FIGO) guidelines [2, 3]. In these positive-risk cases, postoperative adjuvant therapy reduced the local relapse rate and improved the disease-free survival (DFS) and overall survival (OS) time compared with patients treated with surgery only [4–7].

^18^F-2-fluoro-2-deoxy-D-glucose positron emission tomography/computed tomography (^18^F-FDG PET/CT) is an important molecular imaging modality that has been widely used for the diagnosis, staging, response assessment, recurrence detection, and survival analysis of uterine cervical cancer [8]. Among these PRFs, PET/CT is the most commonly used for diagnosing pelvic lymph node metastasis, and the values are variable, with the sensitivity ranging from 8.3 to 82% in early uterine cervical cancer [9–13]. The reason for this inconsistent result may be that lymph node metastasis is not an independent behaviour but is affected by a variety of clinicopathological factors, including tumour differentiation, invasion depth of stroma, involvement of the parametrium, and so on [14]. Moreover, Benedetti-Panici reported that parametrial invasion was through direct extension in 37% of cases, by lymph node metastases in 59% and LVSI in 52% [15, 16]. Therefore, these behaviours were not independent but interactive with each other. Sporadic studies for only one or two particular PRF(s) could not comprehensively reflect the postoperative PRS for recurrence. To date, systematic analyses between pathologic risk status (PRS) and PET/CT have not proceeded.

Since the majority pathological type of cervical cancer is squamous-cell carcinoma, our study was performed in this homologous group of patients with the same histology, which increases the generalizability of our findings. Thus, we intended to comprehensively investigate the correlation between PRS and ^18^F-FDG uptake to determine whether preoperative PET/CT scans can be used to potentially predict the PRS in ECSC.

## Methods and materials

### Patient data

This study was approved by the ethics committee of Fudan University Shanghai Cancer Center. We retrospectively enrolled 240 ECSC patients who underwent PET/CT before radical surgery January 2010 and July 2015. None of the patients received antitumor therapy before surgery. All patients were diagnosed with 2009 FIGO IA2-IIA stage by gynecologic examination. Patient characteristics including age, FIGO stage, preoperative SCCA level, tumor diameter, and PRF were obtained from the medical records.

### PRF and PRS

The pathological diagnoses were reviewed according to WHO criteria by two experienced gynecologic pathologists. According to NCCN and FIGO guideline [2, 3], tumor diameter ≥ 4 cm, stromal invasion depth ≥ 1/2 and/or LVSI were intermediate-risk factors. And parametria involvement, positive surgery margin, and/or pelvic lymph node metastasis were considered as high-risk factors. Patient with at least one of intermediate- and high-risk factors was considered to have intermediate- and high-risk disease. And patient with none of these risk factors was at low-risk disease, patient with at least one was defined as at positive-risk group.

### ^18^F-FDG PET/CT procedure

^18^F-FDG was produced automatically by cyclotron (Siemens CTI RDS Eclipse ST, Knoxville, Tennessee, USA) using the Explora FDG4 module in our center, and the radiochemical purity was over 95%. All patients fasted at least 6 h and the venous blood glucose levels were maintained under 10 mmol/L before ^18^F-FDG injection (7.4 MBq/kg). Patients were required to be quiet after injection for approximately 1 h. Siemens biograph 16HR PET/CT scanner (Knoxville, Tennessee, USA) was performed for scanning. The transaxial intrinsic spatial resolution was 4.1 mm (full-width at half-maximum) in the center of the view. CT scanning (120 kV, 80–250 mA, pitch 3.6, rotation time 0.5) from the proximal thighs to the head was first performed for data acquisition, following by a PET emission scan. The acquisition time was 2–3 min/bed. PET image data sets were iteratively reconstructed using the attenuation correction of CT data, and the infused images were displayed on a workstation. The reconstructed images were then converted to a semiquantitative image corrected by the injection dose and the subject’s body weight (SUV).

### Imaging interpretation

All PET/CT images were evaluated by two experienced nuclear medicine physicians independently and blindly without knowledge of pathologic risk factor. Consensus was reached in case of discrepancy.

A multimodality computer platform (Syngo; Siemens) was used to analyze the ^18^F-FDG PET/CT images. The maximum standardized uptake value (SUVmax) for each patient was calculated by placing a spheroid-shaped volume of interest (VOI) within the cervical tumor. The SUV was calculated as [decaycorrected activity (kBq) per milliliter of tissue volume] / [injected 18F-FDG activity (kBq) per gram of body mass]. Metabolic tumour volume (MTV) and the mean value of SUV (SUVmean) were measured by drawing a contour large enough to encase the primary tumor in the axial, coronal, and sagittal PET images. A usually used threshold in clinical--SUV of 2.5 [16–18] was define as the margins. The voxel was automatically incorporated when the SUV exceeding the threshold of 2.5, then the MTV was produced. The mean voxels within this contouring were measured as SUVmean. The urinary bladder was manually subtracted. Total lesion glycolysis (TLG) was calculated by SUVmean times MTV.

### Statistical analysis

Statistical analyses were carried out using SPSS statistical software (version 21.0; IBM Inc., New York, USA). Continuous variables were described as medians with ranges, and categorical variables were described as frequencies with percentages. The relationships between each PRF and PET/CT were analyzed by T-test. Chi-square for Fisher’s exact test was used for univariate analysis, and binary-logistic regression analysis for multivariate analysis. The Chi-square test in univariate analysis was subjected to 1000 bootstrap resamples by self-sampled method to generalize the result. Receiver operating characteristic (ROC) curve was used to define the optimal cut-off value, and assess the predictive efficiency of the PET/CT parameters for postoperative PRS. The area under the curve (AUC) was used as an indicator for predictive efficiency. All statistical tests were two-sided, and *p* < 0.05 was considered statistically significant.

## Results

### Patient data

Clinicopathologic data and PET/CT parameters of 240 ECSC patients are described in Table 1. The median (range) age was 47.3 (19–74) years old. A total of 140 (58.3%) patients had stage IIA disease. The median (range) tumour diameter and preoperative squamous cell carcinoma associated antigen (SCCA) level were 3.9 (0.2–10.0) cm and 7.00 (0.30–70.00) ng/ml, respectively. Compared with 29 (12.1%) patients in the low-risk group, 202 (84.2%) patients had PRFs, including 201 (83.8%) with intermediate-risk factors, 78 (32.5%) with high-risk factors. In total, 77 (32.1%) patients had both intermediate- and high-risk factors after surgery. For preoperative PET/CT parameters, the median (range) SUVmax, MTV, and TLG values were 13.47 (2.65–71.84), 44.05 (0.28–263.37), and 288.54 (0.76–2183.34), respectively.

**Table 1:** Patient Characteristics (*n* = 240)

Variable	Value
Age (year), median (range)	47.3 (19–74)
Tumor diameter (cm), median (range)	3.9 (0.2–10.0)
SCCA (ng/ml), median (range)	7.00 (0.30–70.00)
2009 FIGO Stage	
IA2-IB2	100 (41.7%)
IIA1-IIA2	140 (58.3%)
Pathologic risk factor	
Low-risk	29 (12.1%)
Positive-risk	202 (84.2%)
Intermediate-risk	201 (83.8%)
High-risk	78 (32.5%)
Both	77 (32.1%)
NA	9 (3.8%)
SUVmax, median (range)	13.47 (2.65–71.84)
MTV, median (range)	44.05 (0.28–263.37)
TLG, median (range)	288.54 (0.76–2183.34)

*Abbreviation*: *SCCCA* squamous cell carcinoma associated antigen, *SUVmax* maximum of standardized uptake value, *MTV* metabolic tumor volume, *TLG* total lesion glycolysis, *NA* not available

### Univariate and multivariate analyses for positive-risk groups

Continuous parameters were dichotomized by the optimal cut-off values derived from ROC curves. The cut-off values of age and SCCA were 50.5 and 2.45, respectively. For PET/CT parameters, the cut-off values of SUVmax, MTV and TLG were 7.96, 20.88, and 137.40, respectively.

**Fig. 1:**
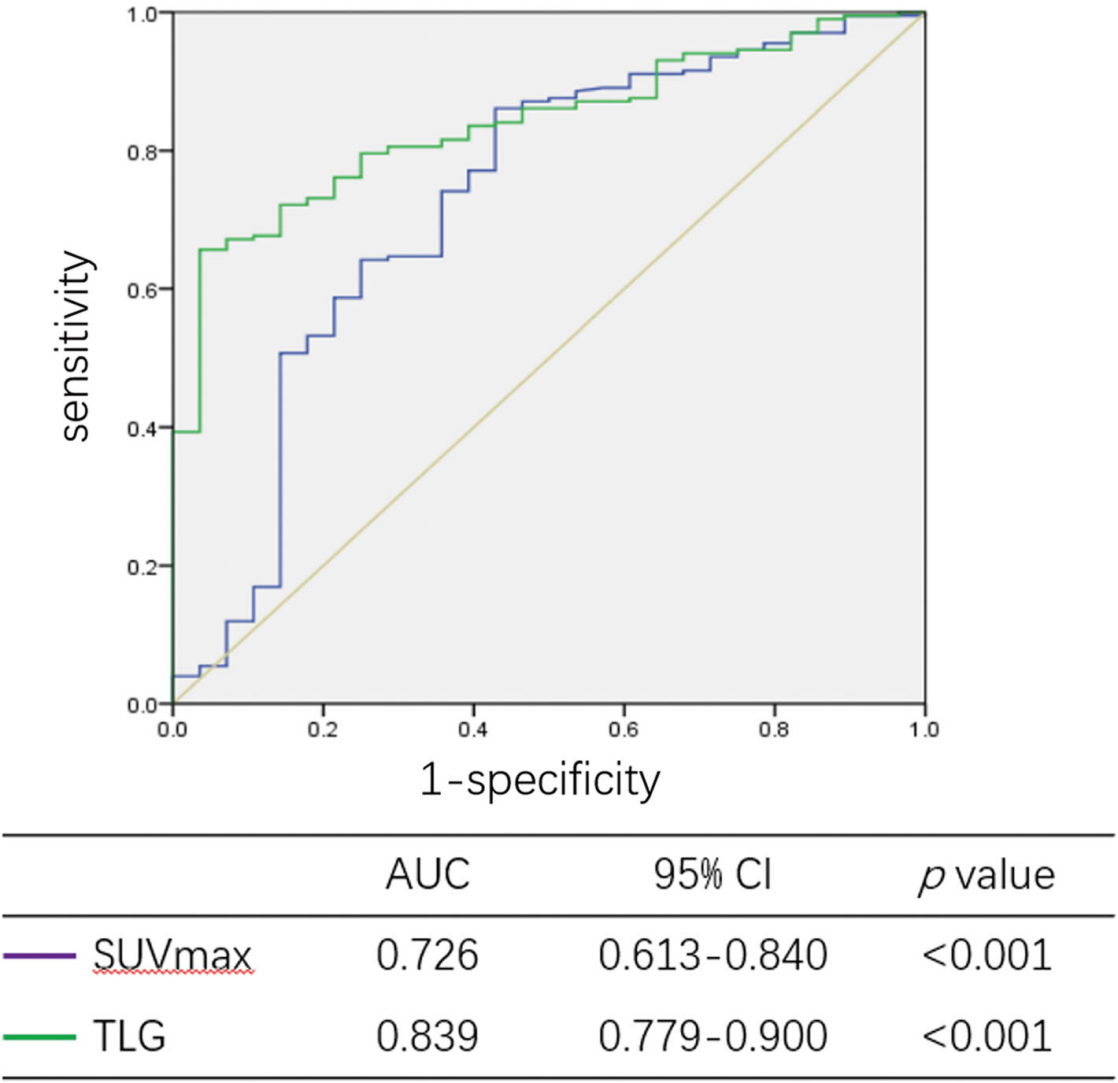
The predictive efficiency of PET/CT for positive-risk status. The AUCs of SUVmax and TLG for the predictive efficiency were 0.726 (95% CI: 0.613–0.840, *p* < 0.001) and 0.839 (95% CI: 0.779–0.900, *p* < 0.001), respectively

In univariate analyses, higher SCCA (*p* < 0.001), FIGO stage (*p* < 0.001), SUVmax (*p* < 0.001), MTV (*p* < 0.001), and TLG (*p* < 0.001) were associated with a higher probability of positive-risk factors. With adjustments for these indicators with statistical significance, SCCA (*p* = 0.005, 95% CI: 1.908–42.182), SUVmax (*p* = 0.029, 95% CI: 1.118–7.746), and TLG (*p* = 0.01, 95% CI: 1.974–132.971) were independent predictors for positive-risk status (Table 2). The AUCs of SUVmax and TLG for the predictive efficiency were 0.726 (95% CI: 0.613–0.840, *p* < 0.001) and 0.839 (95% CI: 0.779–0.900, *p* < 0.001), respectively (Fig. 1).

**Table 2:** Univariate and multivariate analysis for positive-risk disease

Variable	Positive-risk (No. %)		Univariate	Multivariate		
	No	Yes	*p* value	OR	95% CI	*p* value
Age (year)			0.08			
< 50.5	22 (9.5%)	119 (51.5%)				
≥ 50.5	7 (3.0%)	83 (35.9%)				
SCCA (ng/ml)			**< 0.001**	8.972	1.908–42.182	**0.005**
< 2.45	27 (12.1%)	81 (36.2%)				
≥ 2.45	2 (0.9%)	114 (50.9%)				
FIGO stage			**< 0.001**	–	–	0.065
IA2-IB2	21 (9.1%)	72 (31.2%)				
IIA1-IIA2	8 (3.5%)	130 (56.3%)				
SUVmax			**< 0.001**	2.943	1.118–7.746	**0.029**
< 7.96	16 (7.0%)	28 (12.2%)				
≥ 7.96	12 (5.2%)	174 (75.7%)				
MTV			**< 0.001**	–	–	0.426
< 20.88	24 (10.5%)	49 (21.4%)				
≥ 20.88	4 (1.7%)	152 (66.4%)				
TLG			**< 0.001**	16.201	1.974–132.971	**0.01**
< 137.40	27 (11.8%)	69 (30.1%)				
≥ 137.40	1 (0.4%)	132 (57.6%)				

*Abbreviation*: *SCCCA* squamous cell carcinoma associated antigen, *SUVmax* maximum of standardized uptake value, *MTV* metabolic tumor volume, *TLG* total lesion glycolysis

### The details of PRFs and their correlation with PET/CT parameters

The detailed frequencies of PRFs are shown in Table 3. For intermediate-risk factors, excluding the 29 (12.1%) invalid patients, the number of patients with small and large tumours was almost the same (104 (43.3%) versus 107 (44.6%)). Most tumours in 179 (74.6%) patients infiltrated the deep stroma. However, fewer patients (98, 40.8%) had LVSI. For high-risk factors, parametrial invasion occurred in 11 (4.6%) patients, and positive surgery margins occurred in only 3 (1.3%) patients. A total of 74 (30.8%) patients had pelvic lymph node metastasis.

**Table 3:** The detail of PRFs

Variable	Value (No. %)
**Intermediate-risk**	
Tumor diameter (cm)	
< 4	104 (43.3%)
≥ 4	107 (44.6%)
NA	29 (12.1%)
Stromal invasion depth	
< 1/2	54 (22.5%)
≥ 1/2	179 (74.6%)
NA	7 (2.9%)
LVSI	
No	138 (57.5%)
Yes	98 (40.8%)
NA	4 (1.7%)
**High-risk**	
Parametria invasion	
No	225 (93.8%)
Yes	11 (4.6%)
NA	4 (1.7%)
Surgery margin	
Negative	234 (97.5%)
Positive	3 (1.3%)
NA	3 (1.3%)
Pelvic lymph node metastasis	
No	163 (67.9%)
Yes	74 (30.8%)
NA	3 (1.3%)

*Abbreviation*: *LVSI* lymphovascular space invasion, *NA* not available

**Fig. 2:**
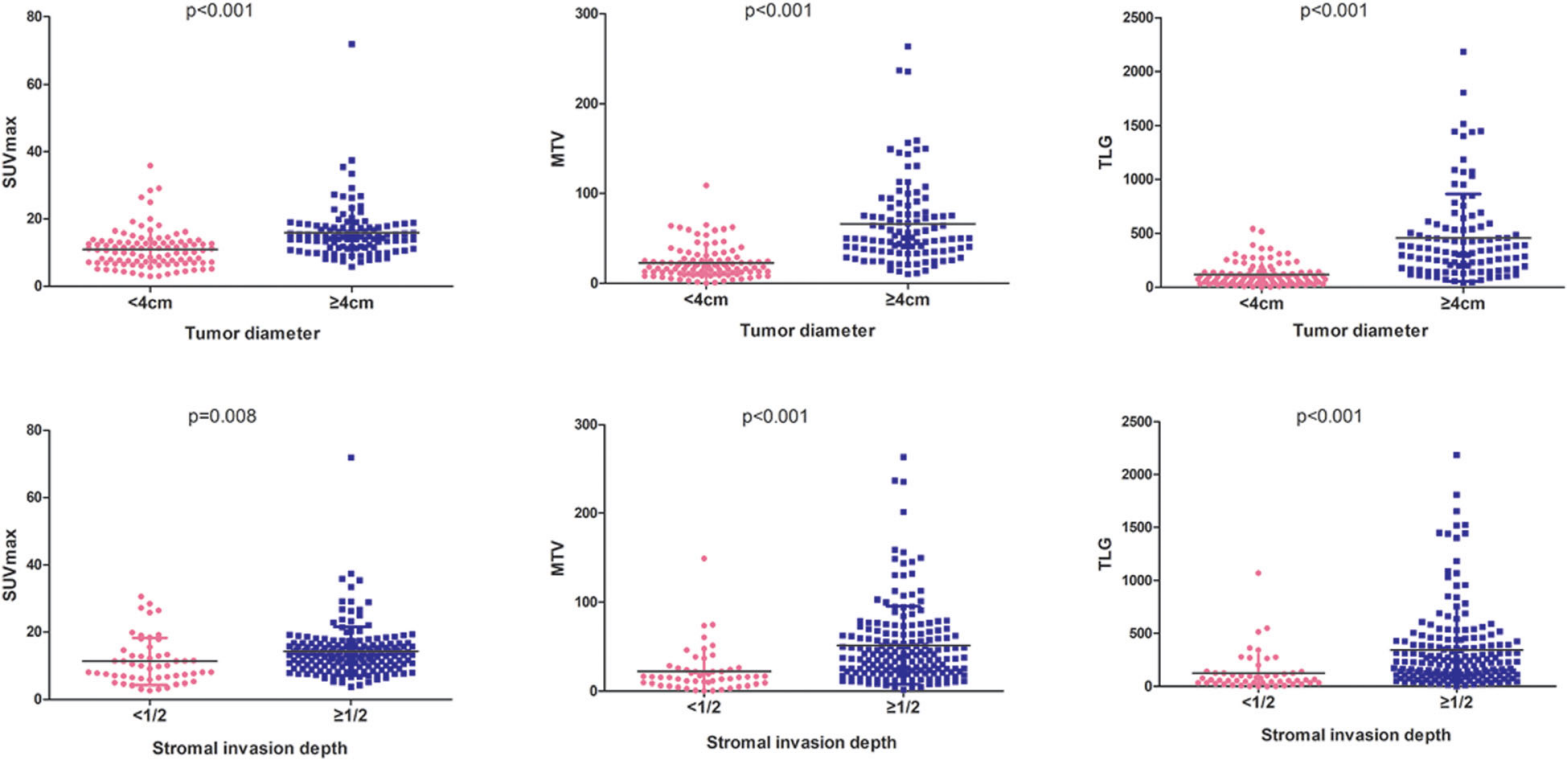
The relationships between ^18^F-FDG uptake and tumor size and deep stromal invasion. All PET/CT parameters including SUVmax, MTV and TLG were associated with tumor size and deep stromal invasion statistically in intermediate-risk disease (all *p* value< 0.001)

**Fig. 3:**
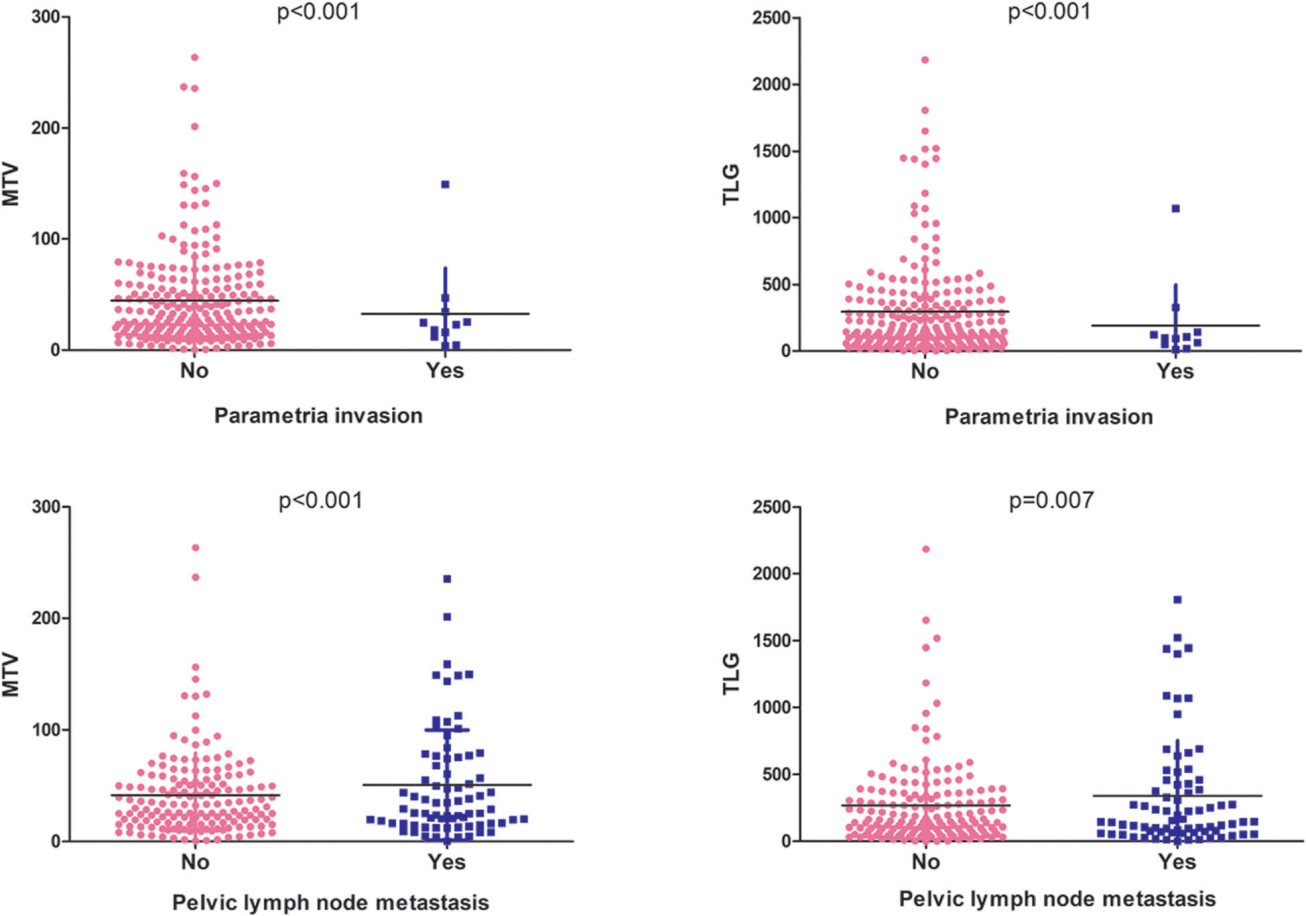
The relationships between ^18^F-FDG uptake and parametria invasion and pelvic lymph node metastasis. For high-risk factor, MTV and TLG had significant relationships with parametria invasion (MTV: *p* < 0.001; TLG: *p* < 0.001) and pelvic lymph node metastasis (MTV: *p* < 0.001; TLG: *p* = 0.007)

We further analysed the relationship between each PRF and PET/CT. The results showed that all PET/CT parameters, including SUVmax, MTV and TLG, were statistically associated with tumour size and deep stromal invasion in the intermediate-risk group (all *p* value< 0.001, Fig. 2). For high-risk factors, MTV and TLG had significant relationships with parametrial invasion (MTV: *p* < 0.001; TLG: *p* < 0.001) and pelvic lymph node metastasis (MTV: *p* < 0.001; TLG: *p* = 0.007) (Fig. 3). However, none of the ^18^F-FDG uptake indicators were associated with the status of LVSI or surgical margin.

### Characteristics of patient with different PRSs

The characteristics of patients at different risk statuses are listed in Table 4. The median (range) age in the three groups was essentially the same: low-risk, 46.3 (29–73) years; intermediate-risk, 47.6 (19–74) years; and high-risk, 47.0 (24–72) years. Tumour size in low-risk patients was smaller than that in intermediate- and high-risk patients (2.2 (0.2–3.8) versus 4.2 (1.1–10.0), 4.5 (1.5–10.0) cm). In the intermediate- and high-risk groups, 107 (53.2%) and 42 (53.8%) patients had larger tumour sizes. The median (range) preoperative SCCA level in low-risk patients was 1.47 (0.40–6.60) ng/ml, which was lower than that in intermediate- (8.04 (0.30–70.00) ng/ml) and high-risk patients (13.13 (0.50–70.00) ng/ml). Compared with 8 (27.6%) patients in the low-risk group, 129 (64.2%) and 50 (54.1%) had stage IIA disease in the intermediate- and high-risk groups, respectively.

**Table 4:** Characteristics of patient with different PRS

Variable	Low-risk (*n* = 29)	Intermediate-risk (*n* = 201)	High-risk (*n* = 78)
Age (year), median (range)	46.3 (29–73)	47.6 (19–74)	47.0 (24–72)
Tumor diameter (cm), median (range)	2.2 (0.2–3.8)	4.2 (1.1–10.0)	4.5 (1.5–10.0)
< 4	29 (100%)	73 (36.3%)	29 (37.2%)
≥ 4	–	107 (53.2%)	42 (53.8%)
NA	–	21 (10.4%)	7 (9.0%)
SCCA (ng/ml), median (range)	1.479 (0.40–6.60)	8.04 (0.30–70.00)	13.13 (0.50–70.00)
2009 FIGO Stage			
IA2-IB2	21 (72.4%)	72 (35.8%)	28 (35.9%)
IIA1-IIA2	8 (27.6%)	129 (64.2%)	50 (54.1%)
SUVmax, median (range)	9.97 (3.03–28.44)	14.08 (2.65–71.84)	13.43 (2.65–37.41)
MTV, median (range)	14.06 (0.28–36.49)	49.15 (0.41–263.37)	61.17 (0.41–263.37)
TLG, median (range)	66.83 (0.76–277.49)	326.69 (1.05–2183.34)	394.56 (1.05–2183.34)

*Abbreviation*: *SCCCA* squamous cell carcinoma associated antigen, *SUVmax* maximum of standardized uptake value, *MTV* metabolic tumor volume, *TLG* total lesion glycolysis, *NA* not available

As for PET/CT scans, patients with low-risk disease showed lower SUVmax, MTV, and TLG than the other two groups. The median (range) details of PET/CT parameters in low-, intermediate- and high-risk disease groups were as follows: SUVmax: 9.97 (3.03–28.44), 14.08 (2.65–71.84) and 13.43 (2.65–37.41); MTV: 14.06 (0.28–36.49), 49.15 (0.41–263.37), and 61.17 (0.41–263.37); TLG: 66.83 (0.76–277.49), 326.69 (1.05–2183.34), and 394.56 (1.05–2183.34), respectively.

### Univariate and multivariate analyses for patients with intermediate- and high-risk factors

**Fig. 4:**
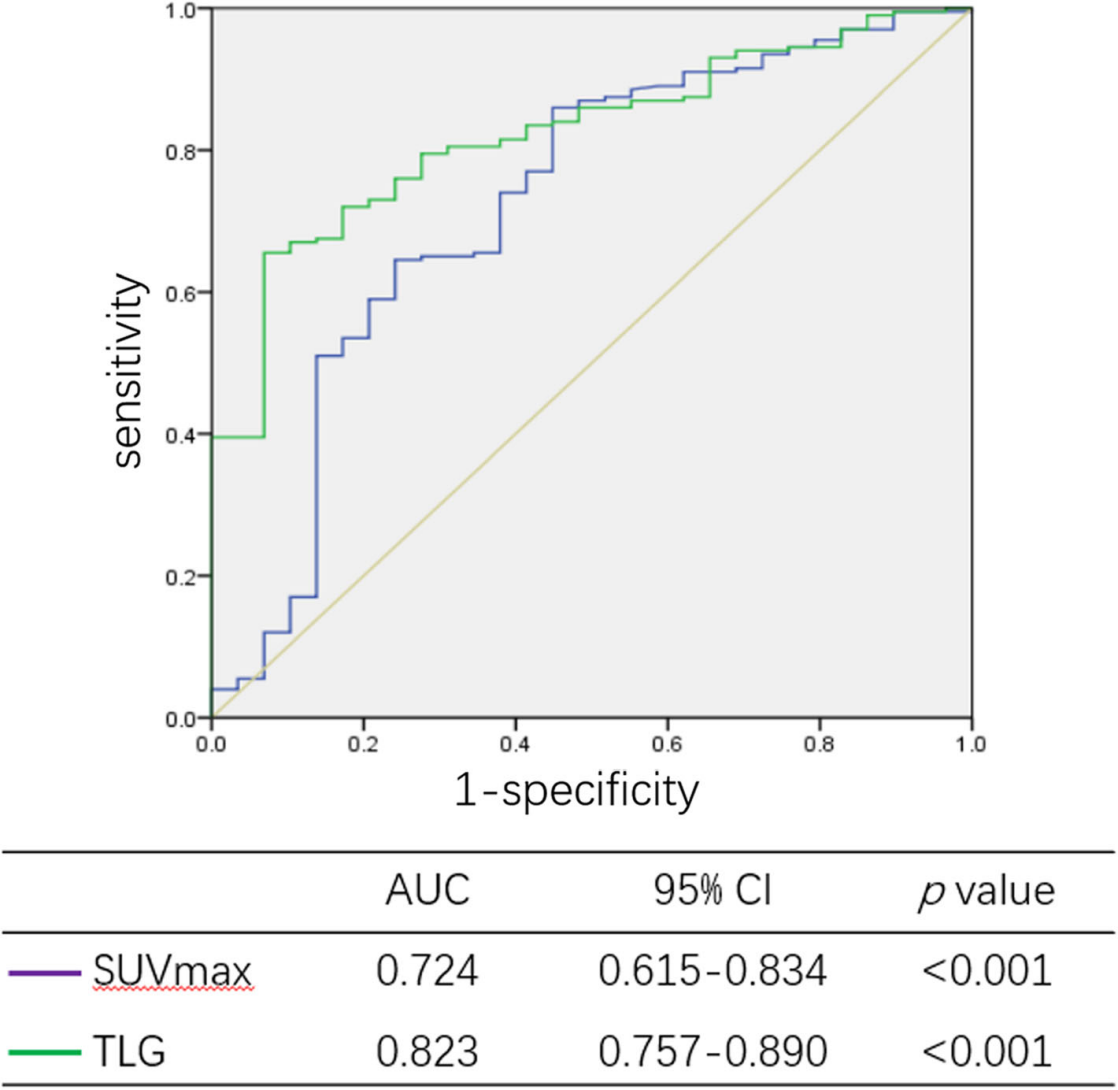
The ROC analysis of PET/CT for intermediate-risk status. The accuracy of SUVmax and TLG for predicting intermediate-risk status were 0.724 (95% CI: 0615–0.834, *p* < 0.001) and 0.823 (95% CI: 0.757–0.890, *p* < 0.001), respectively

In univariate analysis, higher SCCA (*p* < 0.001), FIGO stage (*p* < 0.001), SUVmax (*p* < 0.001), MTV (*p* < 0.001), and TLG (*p* < 0.001) were associated with intermediate-risk patients, and multivariate analyses showed that SCCA (*p* = 0.007, 95% CI: 1.640–22.683), SUVmax (*p* = 0.033, 95% CI: 1.087–7.323), and TLG (*p* = 0.008, 95% CI: 1.770–42.196) were independent predictors for intermediate-risk disease (Table 5). The accuracy of SUVmax and TLG for predicting intermediate-risk status were 0.724 (95% CI: 0615–0.834, *p* < 0.001) and 0.823 (95% CI: 0.757–0.890, *p* < 0.001), respectively (Fig. 4).

**Table 5:** Univariate and multivariate analysis for patient with intermediate-risk factors

Variable	Intermediate-risk (No. %)		Univariate	Multivariate		
	No	Yes	*p* value	OR	95% CI	*p* value
Age (year)			0.139			
< 50.5	22 (9.5%)	119 (51.5%)				
≥ 50.5	8 (3.5%)	82 (35.5%)				
SCCA (ng/ml)			**< 0.001**	6.099	1.640–22.683	**0.007**
< 2.45	27 (12.1%)	81 (36.2%)				
≥ 2.45	3 (1.3%)	113 (50.4%)				
FIGO stage			**< 0.001**	–	–	0.087
IA2-IB2	21 (9.1%)	72 (31.2%)				
IIA1-IIA2	9 (3.9%)	129 (55.8%)				
SUVmax			**< 0.001**	2.821	1.087–7.323	**0.033**
< 7.96	16 (7.0%)	28 (12.2%)				
≥ 7.96	13 (5.7%)	173 (75.2%)				
MTV			**< 0.001**	–	–	0.384
< 20.88	24 (10.5%)	49 (21.4%)				
≥ 20.88	5 (2.2%)	151 (65.9%)				
TLG			**< 0.001**	8.643	1.770–42.196	**0.008**
< 137.40	27 (11.8%)	69 (30.1%)				
≥ 137.40	2 (0.9%)	131 (57.2%)				

*Abbreviation*: *SCCCA* Squamous cell carcinoma associated antigen, *SUVmax* maximum of standardized uptake value, *MTV* metabolic tumor volume, *TLG* total lesion glycolysis

**Fig. 5:**
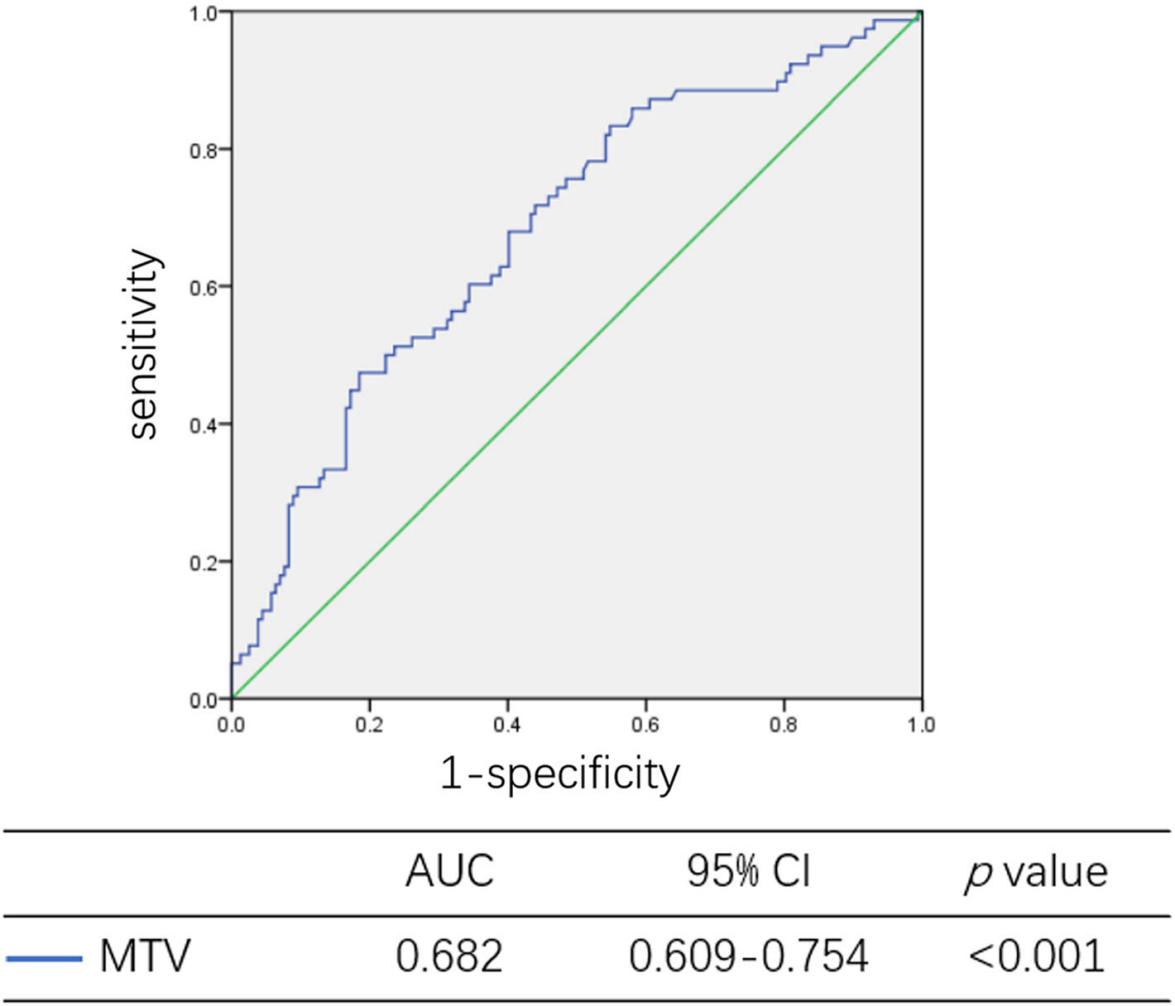
The predictive efficiency of MTV for high-risk status. The predictive efficiency of MTV was 0.682 (95%CI: 0.609–0.754, *p* < 0.001)

For high-risk patients, SCCA (*p* < 0.001), MTV (*p* < 0.001) and TLG (*p* < 0.001) were significant predictive factors in univariate analyses. SCCA (*p* = 0.001, 95% CI: 1.675–6.430) and MTV (*p* = 0.014, 95% CI: 1.230–6.143) were independent of high-risk patients in further multivariate analyses (Table 6). The predictive efficiency of MTV was 0.682 (95% CI: 0.609–0.754, *p* < 0.001) (Fig. 5).

**Table 6:** Univariate and multivariate analysis for patient with high-risk factors

Variable	High-risk (No. %)		Univariate		Multivariate	
	No	Yes	*p* value	OR	95% CI	*p* value
Age (year)			0.580			
< 50.5	96 (40.5%)	50 (21.1%)				
≥ 50.5	63 (26.6%)	28 (11.8%)				
SCCA (ng/ml)			**< 0.001**	3.282	1.675–6.430	**0.001**
< 2.45	95 (41.3%)	18 (7.8%)				
≥ 2.45	62 (27.0%)	55 (23.9%)				
FIGO stage			0.233			
IA2-IB2	70 (29.5%)	28 (11.8%)				
IIA1-IIA2	89 (37.6%)	50 (21.1%)				
SUVmax			0.116			
< 7.96	36 (15.3%)	11 (4.7%)				
≥ 7.96	122 (51.7%)	67 (28.4%)				
MTV			**< 0.001**	2.749	1.230–6.143	**0.014**
< 20.88	66 (28.1%)	11 (4.7%)				
≥ 20.88	91 (38.7%)	67 (28.5%)				
TLG			**< 0.001**	–	–	0.893
< 137.40	81 (34.5%)	20 (8.5%)				
≥ 137.40	76 (32.3%)	58 (24.7%)				

*Abbreviation*: *SCCCA* Squamous cell carcinoma associated antigen, *SUVmax* maximum of standardized uptake value, *MTV* metabolic tumor volume, *TLG* total lesion glycolysis

## Discussion

Our study demonstrated that preoperative ^18^F-FDG PET/CT parameters were independent predictors for the PRS in ECSC patients. Patients had higher SUVmax and TLG in the positive- and medium-risk groups than in the negative- and low-risk groups. For the high-risk status, only MTV was an independent predictor. Thus, we analysed the relationship between each PRF and PET/CT and found that ^18^F-FDG uptake was correlated with tumour size, depth of stromal invasion, parametrial invasion, and pelvic lymph node metastasis in ECSC patients.

The preferred treatment for clinical early-stage patients is radical surgery with or without individualized adjuvant treatment. Adjuvant treatment is indicated to reduce the rate of recurrence after surgery depending on surgical findings and disease stage, which is indicated after radical surgery if PRFs for recurrence are discovered. Clinical reports showed that patients presenting with pathologic intermediate-risk factors, including large tumour size, deep stromal invasion, or LVSI, had a risk of recurrence as high as 30% [4, 5]. Patients with at least one pathologic high-risk factor, including parametria invasion, positive surgical margin, or pelvic lymph node metastasis, were found to have a decreased 5-year survival rate and an increased risk of disease recurrence of 40% [6]. Thus, the NCCN and FIGO guidelines recommend postoperative pelvic radiation with (without) concurrent cisplatin-based chemotherapy for intermediate-risk patients with large primary tumours, deep stromal invasion, and/or LVSI. Pelvic radiation with concurrent cisplatin chemotherapy with (without) vaginal brachytherapy is recommended for patients with high-risk factors, including parametrial invasion, positive surgical margins, and/or pelvic lymph node metastasis. These postoperative interventions have increased the DFS and OS in early-stage cervical cancer patients.

As a functional modality, ^18^F-FDG PET/CT has been widely used in the diagnosis, staging, response assessment, recurrence detection, and survival analysis of uterine cervical cancer [8]. Apart from evaluating pelvic lymph node metastasis, the relationships between ^18^F-FDG uptake and other pathologic risk factors have rarely been explored. Thus, we analysed the relationship between each PRF and PET/CT parameter in our study. The results showed that all PET/CT parameters, including SUVmax, MTV and TLG, were statistically associated with tumour size and deep stromal invasion, which was consistent with previous studies [17, 18]. We also found in PET/CT that parametrial invasion and pelvic lymph node metastasis had significant relationships with MTV and TLG, but not SUVmax, even though magnetic resonance imaging (MRI) is the best technology for evaluating the invasion of the parametrium in uterine cervical cancer. However, none of the ^18^F-FDG uptake indicators were associated with the status of LVSI or a positive surgical margin.

Among these PRFs, PET/CT is most commonly used in the diagnosis of pelvic lymph node metastasis, which is the most important influencing factor for survival and could increase recurrence to 25–30% [4]. However, the diagnostic performances of PET/CT scans are variable, with the sensitivity ranging from 8.3 to 82% in early uterine cervical cancer [9–13]. The unsatisfactory and inconsistent result may be that the metastasis of lymph nodes is not an independent behaviour but is affected by a variety of clinicopathological factors, including tumour differentiation, invasion depth of stroma, involvement of the parametrium, and so on [14]. Moreover, Benedetti-Panici reported that parametrial invasion was through direct extension in 37% of cases, by lymph node metastases in 59% and LVSI in 52% [15, 16]. The interactions among PRFs posed great difficulty in accurately evaluating only one or two particular risk factor(s), hence below the true diagnostic efficiency of a PET/CT examination. Therefore, combined factors should be suggested to better reflect the value of PET/CT in predicting the postoperative recurrent risk status. To date, systematic analysis between PRS and PET/CT has not proceeded. Furthermore, to increase the generalizability of our study, we enrolled the homologous group of patients with squamous-cell carcinoma, which is the most common pathological type of cervical cancer.

In our study, we found that SUVmax and TLG were independent predictors for positive-risk status, and the predictive efficiency was 0.726 and 0.839, respectively. Higher MTV tended to have a higher probability of positive risk factors in the univariate analysis. However, it was not an independent indicator for predicting positive-risk status in the multivariate analysis. Because intermediate- and high-risk factors contribute to different recurrence rates and postoperative adjuvant therapy options, we further evaluated the predictive performance of PET/CT for intermediate- and high-risk status. For intermediate-risk disease, SUVmax and TLG were independent predictors, and the accuracies of SUVmax and TLG for predicting intermediate-risk status were 0.724 and 0.823, respectively. These results were similar to those of positive-risk disease. Roughly the same frequency in our study was the possible reason. In addition, 202 (84.2%) patients had postoperative pathologic risk factors, and 201 (83.8%) had intermediate-risk factors. Only MTV was independently correlated with high-risk disease, even though the predictive value of TLG was significant in the univariate analysis. However, the predictive efficiency of MTV in high-risk disease was not inferior (AUC = 0.682), which might be caused by the low incidence of parametrial invasion, positive surgical margin or lymph node metastasis.

The current study had some limitations. This was a retrospective study with potential recall bias. In addition, although we enrolled a relatively large sample size, the incidence of parametrial invasion and residual microscopic lesion surgical margins were low in early uterine cervical cancer, which might cause possible selection bias. And in this study, we just sought the potential correlations between PET/CT and PFRs, and didn’t pursue the prediction model further by dividing the data into training or testing cohort. Therefore, large defined randomized studies are needed to further characterize the correlation of preoperative PET/CT parameters with high-risk status, and explore the useful prediction model in early-stage cervical cancer. Finally, further studies are needed to determine whether the combination of PET/CT and PRF can improve the predictive accuracy for survival prognosis in ECSC cancer patients.

## Conclusions

In summary, our study demonstrated that preoperative ^18^F-FDG PET/CT had a predictive value for PRS in ECSC noninvasively and independently. Patients had higher SUVmax and TLG in the positive- and medium-risk groups than in the negative- and low-risk groups. For high-risk status, only MTV was an independent predictor. These results may not only help to improve stratification of this patient cohort and make appropriate treatment planning but also extend the application of PET/CT in early-stage cervical cancer.

## Data Availability

Not applicable.

## Abbreviations

PRF: Pathologic risk factor
ECSC: Early-stage uterine cervical squamous cancer
PRS: Pathologic risk status
LVSI: Lymphovascular space invasion
AUC: The area under the curve
SUVmax: Maximum standardized uptake value
MTV: Metabolic tumour volume
TLG: Total lesion glycolysis
ROC: Receiver operating characteristic
SCCA: Carcinoma associated antigen

## References

[1] Bray F, Ferlay J, Soerjomataram I, Siegel RL, Torre LA, Jemal A (2018). Global cancer statistics 2018: GLOBOCAN estimates of incidence and mortality worldwide for 36 cancers in 185 countries. CA Cancer J Clin.

[2] Bhatla N, Aoki D, Sharma DN, Sankaranarayanan R (2018). Cancer of the cervix uteri. Int J Gynaecol Obstet.

[3] Koh WJ, Abu-Rustum NR, Bean S (2019). Cervical Cancer, Version 3.2019, NCCN clinical practice guidelines in oncology. J Natl Compr Cancer Netw.

[4] Sedlis A, Bundy BN, Rotman MZ, Lentz SS, Muderspach LI, Zaino RJ (1999). A randomized trial of pelvic radiation therapy versus no further therapy in selected patents with stage IB carcinoma of the cervix after radical hysterectomy and pelvic lymphadenectomy: a gynecologic oncology group study. Gynecol Oncol.

[5] Rotman M, Sedlis A, Piedmonte MR (2006). A phase III randomized trial of postoperative pelvic irradiation in stage IB cervical carcinoma with poor prognostic features: follow-up of a gynecologic oncology group study. Int J Radiat Oncol Biol Phys.

[6] Peters WA, Liu PY, Barrett RJ (2000). Concurrent chemotherapy and pelvic radiation therapy compared with pelvic radiation therapy alone as adjuvant therapy after radical surgery in high-risk early-stage cancer of the cervix. J Clin Oncol.

[7] Ruengkhachorn I, Therasakvichya S, Warnnissorn M, Leelaphatanadit C, Sangkarat S, Srisombat J (2015). Pathologic risk factors and oncologic outcomes in early-stage cervical cancer patients treated by radical hysterectomy and pelvic lymphadenectomy at a Thai University hospital: a 7 year retrospective review. Asian Pac J Cancer Prev.

[8] Gandy N, Arshad MA, Park WE, Rockall AG, Barwick TD (2019). FDG-PET Imaging in Cervical Cancer. Semin Nucl Med.

[9] Kim DY, Shim SH, Kim SO, Lee SW, Park JY, Suh DS (2014). Preoperative nomogram for the identification of lymph node metastasis in early cervical cancer. Br J Cancer.

[10] Sironi S, Buda A, Picchio M (2006). Lymph node metastasis in patients with clinical early-stage cervical cancer: detection with integrated FDG PET/CT. Radiology.

[11] Signorelli M, Guerra L, Montanelli L (2011). Preoperative staging of cervical cancer: is 18-FDG-PET/CT really effective in patients with early stage disease?. Gynecol Oncol.

[12] Jung W, Park KR, Lee KJ (2017). Value of imaging study in predicting pelvic lymph node metastases of uterine cervical cancer. Radiat Oncol J.

[13] Tanaka T, Sasaki S, Tsuchihashi H, et al. Which is better for predicting pelvic lymph node metastases in patients with cervical cancer: Fluorodeoxyglucose-positron emission tomography/computed tomography or a sentinel node biopsy? A retrospective observational study. Medicine (Baltimore). 2018. 10.1097/MD.0000000000010410. 10.1097/MD.0000000000010410 PMC5916659 29668599

[14] Du R, Li L, Ma S, Tan X, Zhong S, Wu M (2018). Lymph nodes metastasis in cervical cancer: incidences, risk factors, consequences and imaging evaluations. Asia Pac J Clin Oncol.

[15] Benedetti-Panici P, Maneschi F, D'Andrea G (2000). Early cervical carcinoma: the natural history of lymph node involvement redefned on the basis of thorough parametrectomy and giant section study. Cancer.

[16] Dabi Y, Willecocq C, Ballester M, et al. Identification of a low risk population for parametrial invasion in patients with early-stage cervical cancer. J Transl Med DIO. 2018. 10.1186/s12967-018-1531-6. 10.1186/s12967-018-1531-6 PMC6001133 29898732

[17] Xu W, Yu S, Xin J, Guo Q (2016). Relationship of 18F-FDG PET/CT metabolic, clinical and pathological characteristics of primary squamous cell carcinoma of the cervix. J Investig Med.

[18] Chung HH, Nam BH, Kim JW (2010). Preoperative [18F] FDG PET/CT maximum standardized uptake value predicts recurrence of uterine cervical cancer. Eur J Nucl Med Mol Imaging.

